# Measurement of adherence, drug concentrations and the effectiveness of artemether-lumefantrine, chlorproguanil-dapsone or sulphadoxine-pyrimethamine in the treatment of uncomplicated malaria in Malawi

**DOI:** 10.1186/1475-2875-8-204

**Published:** 2009-08-26

**Authors:** David J Bell, Dan Wootton, Mavuto Mukaka, Jacqui Montgomery, Noel Kayange, Phillips Chimpeni, Dyfrig A Hughes, Malcolm E Molyneux, Steve A Ward, Peter A Winstanley, David G Lalloo

**Affiliations:** 1 Tropical and Infectious Diseases Unit, Royal Liverpool University Hospital, Liverpool L7 8XP, UK; 2 Malawi-Liverpool-Wellcome Trust Clinical Research Programme, Blantyre, Malawi; 3 Department of Pharmacology & Therapeutics, University of Liverpool, Liverpool, L69 3GE, UK; 4 Molecular and Biochemical Parasitology Group, Liverpool School of Tropical Medicine, Liverpool, L3 5QA, UK; 5 Centre for Economics and Policy in Health, Bangor University, Bangor, LL57 1UT, UK; 6 School of Clinical Sciences, University of Liverpool, Liverpool, L69 3BG, UK; 7 Clinical Group, Liverpool School of Tropical Medicine, Liverpool, L3 5QA, UK

## Abstract

**Background:**

Sulphadoxine-pyrimethamine (SP) is the only single dose therapy for uncomplicated malaria, but there is widespread resistance. At the time of this study, artemether-lumefantrine (AL) and chlorproguanil-dapsone (CPD), both multi-dose regimes, were considered possible alternatives to SP in Malawi. The aim of this study was to investigate the impact of poor adherence on the effectiveness of AL and CPD.

**Methods:**

Children ≥12 months and adults with uncomplicated malaria were randomized to receive AL, CPD or SP. Adherence was measured using a questionnaire and electronic monitoring devices, MEMS™, pill bottles that recorded the date and time of opening. Day-7 plasma dapsone or lumefantrine concentrations were measured to examine their relationship with adherence and clinical response.

**Results:**

841 patients were recruited. The day-28 adequate clinical and parasitological response (ACPR) rates, using intention to treat analysis (missing data treated as failure), were AL 85.2%, CPD 63.7% and SP 50%. ACPR rates for AL were higher than CPD or SP on days 28 and 42 (p ≤ 0.002 for all comparisons). CPD was more effective than SP on day-28 (p = 0.01), but not day-42.

Very high adherence was reported using the questionnaire, 100% for AL treated patients and 99.2% for the CPD group. Only three CPD participants admitted missing any doses. 164/181 (90.6%) of CPD treated patients took all their doses out of the MEMS™ container and they were more likely to have a day-28 ACPR than those who did not take all their medication out of the container, p = 0.024. Only 7/87 (8%) AL treated patients did not take all of their doses out of their MEMS™ container and none had treatment failure.

Median day-7 dapsone concentrations were higher in CPD treated patients with ACPR than in treatment failures, p = 0.012. There were no differences in day-7 dapsone or lumefantrine concentrations between those who took all their doses from the MEMS™ container and those who did not. A day-7 lumefantrine concentration reported to be predictive of AL treatment failure in Thailand was not useful in this population; only one of 16 participants with a concentration below this threshold (175 ng/ml) had treatment failure.

**Conclusion:**

This study provides reassurance of the effectiveness of AL, even with unsupervised dosing, as it is rolled out across sub-Saharan Africa. Self-reported adherence appears to be an unreliable measure of adherence in this population.

## Background

In 2007, Malawi switched its first line therapy for uncomplicated malaria from sulphadoxine-pyrimethamine (SP) to artemether-lumefantrine(AL, Coartem™), a fixed-ratio combination of artemether (AM) with lumefantrine (LU). The need to take AL twice daily for three successive days means there is a larger potential for poor adherence with AL compared to the single dose regimen of SP.

As part of efforts to optimize the operational use of AL, it is important to assess the effect of poor adherence to (compliance with) this regime on clinical outcome. This paper describes a large single centre open label randomized clinical trial to investigate the effect of adherence upon the effectiveness of AL and chlorproguanil-dapsone (CPD, Lapdap™) in comparison to SP in a 'real-life' setting in adults and children with uncomplicated malaria. The study was initially designed to focus primarily on CPD, an anti-folate combination, previously shown to be effective in Africa in areas with high SP resistance [1]. Subsequent to the completion of the study, CPD was withdrawn by the manufacturers owing to an unacceptably high risk of haemolysis in patients with glucose-6-phosphate dehydrogenase (G6PD) deficiency [2]. Two different methods were used to assess adherence, and the relationship between adherence, day-7 plasma drug concentrations and clinical responses to AL and CPD was examined.

## Methods

### Participants and study site

The study took place at Zingwangwa health centre, in the city of Blantyre, Malawi, where malaria transmission is perennial, peaking during December to April. Between May 2004 and April 2006, adults and children presenting with an illness suggesting falciparum malaria were screened for malaria parasitaemia by a finger-prick thick blood film. Patients willing to enter the trial were additionally screened for eligibility by a questionnaire and capillary blood sample to check their haemoglobin concentration [Hb]. Women ≥12 years had a urine pregnancy test. Those with a positive blood film were eligible for enrolment in the study if they fulfilled the following criteria: i) age ≥6 months, ii) weight ≥10 kg, iii) *P. falciparum *monoinfection on the blood slide, iv) Hb ≥7 g/dl, and v) no features suggesting severe malaria or a concomitant illness. Pregnant or breastfeeding women, women with a positive pregnancy test and patients with known G6PD deficiency or allergies to sulphonamide or dapsone were excluded. Written informed consent was required from the participant, or from the parent or guardian in the case of children. Approval was obtained from the research ethics committees of the College of Medicine, University of Malawi and Liverpool School of Tropical Medicine. A data and safety monitoring board was appointed. The study was registered with the Clinical Trials register, ISRCTN 12285821.

### Randomization and treatment

Patients meeting inclusion criteria were recruited and randomized to AL, CPD or SP on a 1:2:1 basis. Randomization was performed in blocks of 20 using an off-site computer-generated code. Sealed sequentially numbered envelopes designating treatment group were opened for each participant following entry to the trial.

CPD was dosed by height; paediatric and adult tablet formulations were available. A total of three doses of CPD were prescribed, one daily for three days. AL was dosed by weight and six doses were prescribed; on recruitment and after 8, 24, 36, 48 and 60 hours. The single SP dose was based upon age. The dosing schedules are shown in Table 1.

**Table 1:** Dosing schedules for chlorproguanil-dapsone (CPD), artemether-lumefantrine (AL) and sulphadoxine-pyrimethamine (SP)

**CPD Paediatric Tablets^1^**		**CPD Adult Tablets^2^**		**AL^3^**		**SP^4^**	
**One dose, once daily for 3 days**		**One dose, once daily for 3 days**		**One dose, twice daily for 3 days**		**Single dose**	
**Height** **(cm)**	**No. of Tablets per dose**	**Height** **(cm)**	**No. of Tablets per dose**	**Weight** **(kg)**	**No. of Tablets per dose**	**Age (years)**	**No. of Tablets per dose**
**50 - 64**	0.5	**115 - 134**	0.5	**10 - 14**	1	**0.5 - <2**	0.5
**65 - 84**	1	**135 - 164**	1	**15 - 24**	2	**2 - 8**	1
**85 - 104**	1.5	**≥ 165**	1.5	**25 - 34**	3	**9 - 14**	2
**105 - 114**	2			**≥ 35**	4	**≥ 15**	3

^1^Paediatric CPD tablets contained 15 mg of CPG and 18.75 mg of DDS.

^2^Adult CPD tablets contained 80 mg of CPG and 100 mg of DDS.

^3^AL tablet contained 20 mg of AM and 120 mg of LU.

^4^SP tablets contained 500 mg of sulphadoxine and 25 mg of pyrimethamine.

The first dose of medication was given at the clinic and further doses of CPD and AL were taken at home. Verbal instructions on how to take the remaining doses were given with advice to take AL with food or milk. A three-day supply of paracetamol (10 mg/kg) was given to take as required. Participants were cautioned against taking other anti-malarial drugs. Participants were observed for one hour to ensure that the medication was not vomited: if vomiting occurred within the first hour, the dose was repeated. Vomiting of the repeated first-dose resulted in removal from the trial, treatment with parenteral quinine and transfer to hospital for assessment.

### Classification of outcomes

Formal follow up took place on days 7, 14 and 28 but participants could present to the clinic on any other day if unwell. In the second half of the study, follow up was extended to day-42 to detect treatment failures occurring after 28 days. Patients who did not attend for follow up were visited at home by study personnel (subject to consent) to ensure their safety. At each follow up visit, symptom histories were taken, Hb was checked and a malaria blood slide examined. In addition, on day-7, a venous blood sample was collected for pharmacokinetic analysis (see below). Clinical outcome was assessed using modified World Health Organization (WHO) criteria [3]. Modification was necessary owing to the lack of scheduled follow up on days 2 and 3. Participants were withdrawn if they failed to attend for follow up or withdrew consent.

### Measurement of adherence

Adherence with the SP regimen was assured by direct observation of the single dose therapy. Adherence with AL and CPD was measured in two ways:

1. Use of simple drug dosing questionnaires on day-7; participants or guardians were asked how many tablets had been taken and when.

2. Use of MEMS™ (Aardex Ltd, Switzerland) capped pill bottles which recorded electronically the date and time when the medication bottle was opened. Adherence was estimated by comparing the MEMS™ number of openings with the prescribed dosing regime. MEMS™ were used in a sub-group of patients according to availability at the time of recruitment. The MEMS™ data were interpreted as follows; for CPD, participants who opened their pill bottle one time or more on each of the two consecutive days after recruitment were assumed to have taken all their prescribed doses of medication. Those who failed to open their pill bottles on the two consecutive days after recruitment were assumed to have missed some doses. For AL, if the MEMS™ recorded at least one opening of the bottle on the day of recruitment and at least two more on each of the next two days, it was assumed that all the doses were taken.

### Laboratory methods

Hemocue^® ^Hb estimates and malaria slides were performed from capillary blood sample at each follow up visit. Blood slides were stained with Field's stain and parasite densities estimated from thick films by counting the number of parasites per 200 white blood cells assuming a total count of 8,000/μl. The concentrations of dapsone (DDS) and lumefantrine (LU) were measured from venous blood collected on day-7 to determine whether they were associated with treatment compliance or outcome. The blood was spun at 2,000 g for 10 minutes in a refrigerated centrifuged and the plasma removed and stored at -80°C for the pharmacokinetic analyses.

Dapsone (DDS) was quantified from 0.5 ml of plasma using HPLC-UV (high performance liquid chromatography - ultra-violet) detection using a method developed and validated in Liverpool (SA Ward, unpublished). Chromatographic separations were carried out using Surveyor HPLC system (Thermo Fisher Scientific). Internal standard (pyrimethamine, 500 ng) was added to the plasma samples along with 0.5 ml sodium hydroxide (0.1 M). The samples were then vortexed for 10 seconds followed by the addition of 5 ml dichloromethane and again vortexed for a further 20 seconds. The samples were then centrifuged at 1,600 g for 10 minutes after which the organic (bottom) layer was transferred to a clean tube. A further 5 ml of dichloromethane was added to the aqueous layer, vortexed for 20 seconds and centrifuged again at 1,600 g for 10 minutes with the organic layer collected once more. The pooled organic layers were evaporated to dryness under a steady stream of nitrogen at 30°C. Samples were reconstituted in 100 μl of mobile phase of which 50 μl was injected onto column.

Chromatographic separation was achieved using a Phenomenex Synergi 4 μ MAX RP C12 (150 cm × 4.6 mm, 4 μm particle size) column, fitted with a LiChrosphere^® ^100Rp-18, 5 μ guard column. The mobile phase comprised of acetonitrile: triethylamine (0.1% adjusted to pH 3 with ortho-phosphoric acid): methanol (7:70:23 v/v), flowing at 1 ml/min. Column and tray temperature control was set at room temperature. The UV detector was set at 254 nm. Data was captured and processed using Xcalibur 1.4 software (Thermo Fisher Scientific). The lower limit of quantification (LLOQ) of the dapsone assay was 100 ng/ml and the calibration curve was linear in the range 0-2500 ng/ml.

Lumefantrine was quantified from 0.25 ml of plasma using HPLC-UV detection following a previously described method [4]. The LLOQ of the LU assay was 0.025 ng/ml and the calibration curve was linear in the range 0-20 μg/ml.

### Statistical methods

The primary aim of the study was to investigate the effect of incomplete adherence on the effectiveness of AL and CPD. Effectiveness was assessed by comparing the day-28 "adequate clinical and parasitological response" (ACPR) rates. The ACPR rates were not corrected using the polymerase chain reaction (PCR) to distinguish recurrent parasitaemias due to reinfections and recrudescence (true treatment failures). Secondary aims were to compare the effectiveness of AL, CPD and SP on days 28 and 42, to compare the different methods for measuring adherence, to investigate the associations between day-7 drug concentrations and adherence and treatment outcome and to compare serious adverse events (SAEs) and Hb changes after the three treatments.

An approximate sample size of 1,000 participants was calculated, with 500 participants in the CPD group and 250 in the SP and AL groups. Assuming 50% of the participants were fully adherent, this sample size would detect a difference between a cure rate of 97% in the CPD arm and 90% in the SP arm with 95% confidence and a power of 80%. Because the initial focus of the study was on CPD, we planned to recruit twice as many children into that treatment group than the SP or AL groups. Data were double entered and validated prior to the analyses.

Data analysis was performed using Stata 8: the primary analysis was on an intention to treat (ITT) basis. Binomial regression was used to obtain risk differences between treatments and 95% confidence intervals. Fisher's exact p-values were reported. Tests of significance were performed using the 0.05 level to infer significance for the planned analyses. For pair wise comparisons between combination therapies, we adjusted the significance level to 0.017 (i.e. 0.05/3) using Bonferroni's approach.

## Results

### Recruitment and participant flow

**Figure 1:**
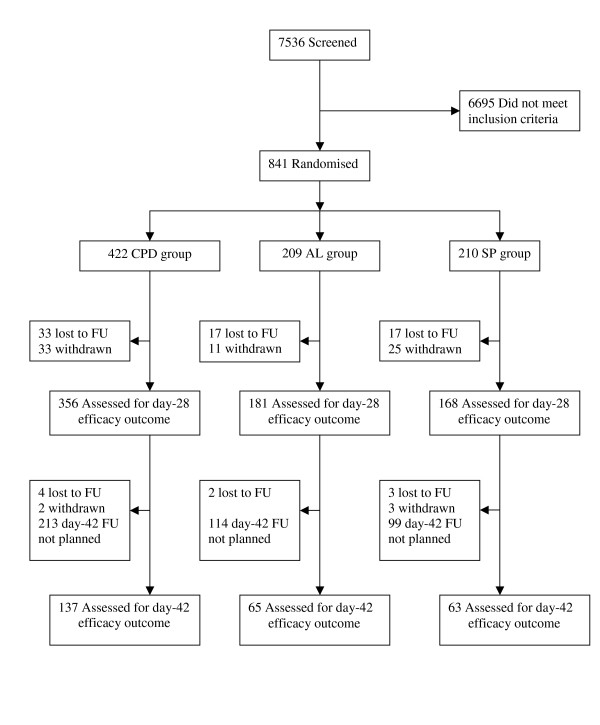
**Study profile (FU = Follow up)**.

841 of the 7,536 patients screened met the inclusion criteria and were enrolled. Common reasons for non-recruitment included negative blood slides (n = 6150), Hb <7 g/dl (n = 189) and the child weighing <10 kg (n = 63). Baseline characteristics of the recruited patients are shown in Table 2. 99 (11.8%) patients had been withdrawn from the study by day-14 and 136 (16.2%) by day-28. Recruitment was extended to 42 days for the last 330 patients enrolled; 20% of these were withdrawn by day-42 (Figure 1).

**Table 2:** Baseline characteristics by treatment group

**Variable**	**Statistic**	**Treatment Group**		
		**CPD**	**AL**	**SP**
**Number of patients**		422	209	210
				
**Age (Years)**	**Median**	10.3	8.9	10.6
	**IQR**	18.1	15.7	17.8
**<5 years**		129	57	53
				
**Sex**	**Female (%)**	193(45.7)	89 (42.6)	82 (39.1)
				
**Weight (kg)**	**Median**	27	24	27.5
	**IQR**	38	36	38
				
**Temperature (°C)**	**Mean**	37.6	37.8	37.5
	**S.D.**	1.4	1.5	1.4
**> 37.5°C**	**Number**	203	115	89
				
**Parasite count (/μl)**	**Mean**	16263	21262	13489
	**Range**	13 - 207000	20 - 258750	23 - 227500
				
**Haemoglobin (g/dl)**	**Mean**	11.3	11.1	11.6
	**s.d**	2.1	2.1	2.3
	**Missing**	18	6	5

IQR = Inter-quartile range

S.D. = Standard Deviation

### Treatment effectiveness

The results of the day-28 and day-42 ITT analyses with missing outcomes treated both as failures and successes and the per protocol (PP) analyses are shown in Tables 3 and 4 respectively. In all analyses, the ACPR rates with AL were significantly higher than the rates in either of the other groups on days 28 and 42 (p ≤ 0.002 for all comparisons). The ACPR rates using CPD were significantly higher than SP on day-28 but not on day-42.

**Table 3:** Summary of the Day-28 ACPR rates and difference (95% confidence intervals), the data is not PCR corrected.

**Day-28 Outcome**	**ACPR Rate**	**ACPR Difference (95% Confidence Intervals)**	**P value**
**ITT (missing = failure)**			
**CPD (n = 422)**	63.7%		
**AL (n = 209)**	85.2%		
**SP (n = 210)**	50.0%		
**CPD vs SP**		13.7% (5.6%, 21.9%)	0.001
**AL vs CPD**		21.4% (14.8%, 28.1%)	< 0.001
**AL vs SP**		35.2% (26.9%, 43.5%)	< 0.001
**ITT (missing = success)**			
**CPD (n = 422)**	79.4%		
**AL (n = 209)**	98.6%		
**SP (n = 210)**	70.5%		
**CPD vs SP**		9.4% (2.1%, 16.7%)	0.01
**AL vs CPD**		19.2% (15.0%, 23.4%)	< 0.001
**AL vs SP**		28.6% (22.2%, 35.0%)	< 0.001
**Per Protocol**			
**CPD (n = 356)**	75.6%		
**AL (n = 181)**	98.3%		
**SP (n = 168)**	62.5%		
**CPD vs SP**		13.1% (4.5%, 21.6%)	0.003
**AL vs CPD**		22.8% (17.9%, 27.6%)	< 0.001
**AL vs SP**		35.8% (28.3%, 43.4%)	< 0.001

**Table 4:** Summary of the Day-42 ACPR rates and difference (95% confidence intervals), the data is not PCR corrected

**Day-42 Outcome**	**ACPR Rate**	**ACPR Difference (95% Confidence Intervals)**	**P value**
**ITT (missing = failure)**			
**CPD (n = 166)**	55.4%		
**AL (n = 82)**	75.6%		
**SP (n = 82)**	46.3%		
**CPD vs SP**		9.1% (-4.1%, 22.3%)	0.224
**AL vs CPD**		20.2% (8.2%, 32.2%)	0.002
**AL vs SP**		29.3% (15.0%, 43.5%)	< 0.001
**ITT (missing = success)**			
**CPD (n = 166)**	72.9%		
**AL (n = 82)**	96.3%		
**SP (n = 82)**	69.5%		
**CPD vs SP**		3.4% (-8.7%, 15.4%)	0.653
**AL vs CPD**		23.4% (15.6%, 31.3%)	< 0.001
**AL vs SP**		26.8% (16.1%, 37.6%)	< 0.001
**Per Protocol**			
**CPD (n = 137)**	75.6%		
**AL (n = 65)**	98.3%		
**SP (n = 63)**	62.5%		
**CPD vs SP**		6.8% (-7.6%, 21.3%)	0.425
**AL vs CPD**		28.2% (18.9%, 37.6%)	< 0.001
**AL vs SP**		35.1% (22.0%, 48.2%%)	< 0.001

### Haemoglobin changes

Table 5 shows the mean changes in Hb after treatment with CPD and AL compared to the changes in the SP treatment group on days 7, 14 and 28. No differences in mean Hb level were observed after treatment with CPD or SP. Hb initially fell in both groups below the recruitment level by day-7 but recovered by day-28 to levels above those on day 0. After treatment with AL, the Hb rose by day-14 and 28; this rise was significantly greater than the change after SP.

**Table 5:** Mean changes in Haemoglobin [Hb] after treatment with CPD and AL compared to the Hb changes in the SP treatment group on days 7, 14 and 28.

	**Treatment Group**	**Mean Hb** **(g/dl)**	**Contrast with SP group**		
			**Mean Difference**	**95% confidence interval**	**P-value**
**CPD**					
**Day Number**	**Day-7**	10.42	-0.32	-0.57, -0.08	0.01
	**Day-14**	10.83	-0.03	-0.47, 0.41	0.9
	**Day-28**	11.29	0.12	-0.18, 0.42	0.45
					
**AL**					
**Day Number**	**Day-7**	11.05	0.32	0.04, 0.60	0.03
	**Day-14**	11.31	0.44	-0.06, 0.94	0.09
	**Day-28**	11.81	0.64	0.30, 0.98	< 0.001

### Safety and tolerability

There were 11 serious adverse events (SAEs) in the study, including one death, with eight SAEs in the CPD group and three in the SP treatment group. SAE rates are similar as 422 patients were recruited to the CPD group and 210 to the SP group. SAEs were defined as any occurrence resulting in death or admission to hospital for participants under follow up in the study. The three-year old girl who died had a recruitment parasite count of 129,000 per μl, Hb 11.7 g/dl and packed cell volume (PCV) 34%. She received her first dose of CPD in the clinic. The following day, she had a convulsion and further fevers and on admission to hospital was found to a positive malaria blood slide, PCV 30% and normal blood glucose concentration. She was treated with intravenous quinine, chloramphenicol and penicillin. Convulsions continued despite treatment with lorazepam and she died two days after her admission to hospital with presumed cerebral malaria.

SAEs in the seven remaining CPD treated patients included two patients (aged 8 and 20 years) who developed vomiting and abdominal pain and two adults admitted with fever one week after recruitment and found to have *Salmonella typhimurium *bacteraemia (one was confirmed HIV positive, the other was not tested). One patient developed features of severe malaria (convulsions and fever with a positive blood slide) 20 days after treatment with CPD, having had negative slides on days 7 and 14. Two patients developed severe anaemia within a week of treatment with CPD and required admission to hospital for blood transfusion. The first was a 30-year male, recruited with a Hb of 8.9, who complained of dizziness but no fever on day-7 and had a Hb of 4.8 g/dl and a negative malaria slide. The second, an 11 year boy, was recruited with a Hb of 11.6 g/dl (PCV 34%), but admitted to hospital on day-3 with vomiting, a PCV of 21% and a negative malaria slide. By day-4 the PCV had fallen to 13% (malaria slide still negative) necessitating a blood transfusion. Neither patient became jaundiced, clinically or biochemically, or had a palpable spleen. Three patients taking SP aged 2, 4 and 15 years had SAEs, being admitted to hospital on days 1, 2 and 10 respectively with fever and found to have positive malaria blood slides. All three were diagnosed with severe malaria and received parenteral quinine before discharge. The 2 year old child required blood transfusion due to a fall in their PCV from 27% to 13% by day-2.

### Adherence with AL and CPD treatment regimes

#### Dosing questionnaire

This was completed by 371/442 (88%) participants who took CPD and 185/209 (88.5%) who took AL. Missing data was due to patient withdrawals from the study or loss to follow up. All 185 participants in the AL group who completed questionnaires said that they had taken all six of their prescribed doses. Only three (0.8%) CPD participants admitted missing any doses.

#### MEMS™ results

Data were available for 181 patients in the CPD arm; 164 (90.6%) took all their doses out of the MEMS™ container while 17 (9.4%) did not. Participants who took all their doses were more likely to have an ACPR on day-28, p = 0.024 (Fishers exact). MEMS™ data were available for 87 AL treated patients; 80 (92%) took all their doses out of the MEMS™ container and seven (8%) did not. None had treatment failure by day-28 and so the association between the MEMS™ data and day-28 outcome could not be tested.

#### Day-7 dapsone concentrations

Day-7 PK samples were analysed from 348/422 (82.5%) of the CPD patients. Missing data were due to patient withdrawals before day-7 and samples not collected or lost. The DDS concentration in their day-7 sample was < LLOQ of the assay in 174 (50%) of the participants. Participants whose day-7 DDS concentration was < LLOQ were younger, shorter in height and received a lower dapsone dose per kg body weight (p ≤ 0.001 for all) and were less likely to have an ACPR outcome on day-28 than those ≥ LLOQ (104/178 (59.8%) vs. 143/174 (82.2%), p < 0.0001). For the 174 (50%) participants with quantifiable DDS concentrations, median day-7 DDS concentrations were higher in the ACPR group than the treatment failure group, 248.3 ng/ml vs. 152.3 ng/ml respectively, p = 0.012 (Mann-Whitney). There was no difference in the median day-7 DDS concentration between participants who took all their CPD doses and those who did not according to the MEMS™.

#### Day-7 lumefantrine concentration

Day-7 samples was analysed for LU in 167/209 (79.9%) AL patients. Only 4/167 (2.4%) participants had day-7 LU concentrations < LLOQ of the assay. The median (IQR) LU concentrations for the 163 participants with quantifiable LU concentrations were 214 ng/ml (118-321). The small numbers of treatment failures or patients with samples < LLOQ does not permit meaningful comparisons of lumefantrine levels in these groups. The median (IQR) day-7 LU concentration in the 61 patients who took all their doses out of the MEMS™ container was 193.0 ng/ml (105.0 - 273.0), compared to 127.0 ng/ml (42.0 - 390.0) in the five who did not, (p = 0.264, Mann-Whitney).

A previous study from Thailand reported that patients with LU levels <175 ng/ml on day-7 are more likely to experience recrudescence by day-42 [5]. In this study, of the 58 participants in the AL group with day-42 efficacy data, 16 (27.6%) had day-7 LU levels below this threshold. Only one of these patients went on to have treatment failure by day-42.

## Discussion

Adherence to therapy is one of the cornerstones of successful treatment and the more complex a treatment regime is, the more likely it is that patients will fail to adhere properly. Single dose therapy - SP is the only example - is ideal, but widespread resistance means that SP is no longer a useful option for control programmes. All of the current treatment regimes promoted by the WHO are multi-dose and there is no single dose therapy on the horizon. The impact of poor adherence on treatment effectiveness is difficult to measure but is an important consideration for health policy makers. Methods for measuring adherence to therapy including drug questionnaires, pill counting, assessment of pharmacy repeated prescriptions, MEMS™ and drug level monitoring. None of these is perfect, and measurement of adherence may itself lead to a change in adherence behaviour of the individual being monitored [6].

AL is currently the most widely deployed artemisinin-combination therapy (ACT) in the world. By the end of 2008, it had been adopted as first line treatment policy for uncomplicated malaria in 23 countries in Africa [7]. Treatment with AL requires two doses daily for three consecutive days, and CPD requires a single dose daily for three consecutive days. In this study, two methods to measure patient adherence to AL and CPD treatment, a dosing questionnaire and MEMS™. The simplest of these measures, the drug questionnaire, is the one most open to patient bias. The reported 100% adherence with AL and 99.2% adherence with CPD disagreed with the MEMS™ adherence data from the same patients. This discrepancy between self-reported adherence and electronically measured adherence has been previously described, suggesting that this method is an inaccurate way of assessing adherence [8]. Patients may be keen to appear to be adherent and so unwilling to report missed doses. A similar pattern of very high reported adherence to anti-retroviral therapy (ART) using questionnaires has been noted previously in HIV positive patients in Malawi [9].

MEMS™ provide a more objective assessment of adherence, although the interpretation of their results can be difficult. They have been used extensively to monitor patients on long-term treatment such as anti-retroviral therapy. MEMS™ record the date and time at each opening of the pill bottle. The numbers of tablets removed each time or whether the tablets are actually taken cannot be recorded. Patients taking AL may open the bottle once in the morning and remove all the tablets required for both the morning and evening doses at that time and this would be interpreted as poor adherence due to the missed evening dose. Patients are also known to "play" with their pill bottles and, as a result, multiple openings are recorded each day when no drug has actually been taken.

In this study, over 90% of the patients receiving CPD took all their doses out of the MEMS™ container and these patients were more likely to have an ACPR on day-28 than those participants who did not, p = 0.024. For AL, the MEMS™ data suggested that 92% of the patients took all of their six doses. There were only three treatment failures in the AL treatment group by day-28 making it impossible to examine the association between the MEMS™ results and treatment response.

The median day-7 LU concentration reported here was 214 ng/ml, after a mean total LU dose of 63.9 mg/kg. This is lower than the median of 528 ng/ml reported by Price *et al *after a mean total dose of 61.2 mg/kg of LU [5]. This variability in LU concentration may reflect a number of factors including whether the tablets were taken with fatty foods (to improve LU absorption), and differences in the disposition of the drug between individuals of different age, sex, weights and other factors. This study was not powered or designed to investigate all of these possible causes of variability. Differences in patient adherence (number of tablets taken) may explain some of the differences between these studies. In this study only the first dose of AL was supervised, while in the Price study all doses were supervised. However in this study, only five patients in the AL group took less than their full treatment course according to the MEMS™ and there was no statistical difference between the LU concentrations in these patients compared to those who took all their tablets.

Other studies have addressed adherence with AL. In Bangladesh, an adherence rate of 93% was reported using pill counting and questionnaires and there was no difference noted in day-7 LU concentrations in poorly adherent patients [10]. Average adherence rates of 94% to AL have been reported from a series of home treatment studies in children from Uganda and Ghana [11]. In these studies, adherence was assessed using interviews with the parents of the children. Finally, a study from Uganda reported adherence levels of 90% using pill counting and questionnaires and mean day-3 LU concentrations were lower in non-adherent than adherent patients [12].

The measurement of the day-7 drug concentration has been proposed as a way to predict treatment outcome in malaria treatment studies [13]. A study from Thailand using AL for uncomplicated malaria demonstrated that patients with plasma LU levels below 175 ng/ml on day-7 were more likely to experience recrudescence by day-42 [5]. However, this cut-off was not associated with day-42 treatment failure in this study; 16 (27.6%) patients had concentrations below this threshold and only one of these had a treatment failure and illustrating that drug concentration thresholds, predictive of treatment response in SE Asia, may not be appropriate for use in Africa.

Malawi was the first country in Africa to introduce SP as its first line treatment for uncomplicated malaria in 1993. In 2007, it was withdrawn by the national Malaria Control Programme, and replaced by AL. The results reported here confirm that SP was insufficiently effective for a first-line malaria treatment in Malawi at the time of this study; the day-28 ACPR rate for SP of 62.5% by PP analysis. In contrast, the PP day-28 and day-42 ACPR rates for AL were 98.3% and 95.4% respectively, in keeping with rates reported from other countries in the region including Mali, Ghana and Uganda, whether AL is taken supervised in an efficacy trial or unsupervised [14-16]. These results show that treatment appears to be successful even in the few patients who did not take all their tablets out of their MEMS™ container, suggesting that AL was "forgiving", still having a therapeutic effect in individuals who have missed doses [17].

The PP day-28 ACPR rate with CPD was 75.6% and was lower than had been expected. A longitudinal study from Malawi in 1999 reported a day-14 efficacy of around 96% [1]. Some of this difference may be due to poor adherence; the MEMS™ data showed that 9.4% of the patients did not take the full course of CPD and that these patients were more likely to fail treatment. PCR correction for reinfection would also have increased this ACPR rate; no attempt was made to distinguish reinfections from recrudescent malaria, because from a programmatic viewpoint, both of these are important components of the malaria burden in a population. The poor CPD efficacy might also have been due to increased parasite resistance.

There were no SAEs in the AL treatment group, consistent with data from other studies reporting that this combination therapy is well-tolerated and highly efficacious. Three patients in the SP group required hospital admission with features of severe malaria within 10 days of recruitment to the study, probably reflecting the poor efficacy of SP. One child died having developed features of severe malaria a day after recruitment and treatment with CPD; it is impossible to know whether this resulted from poor treatment efficacy or was an inevitable consequence of that child's infection. The production of CPD was halted and the drug withdrawn because of concern about haemolysis. Two patients developed severe anaemia within a week of treatment with CPD. In both, the Hb fell by around 50% despite parasite clearance, making it unlikely that malaria was responsible. Dapsone-induced haemolysis is more common in G6PD deficient individuals but the G6PD status of participants in this study is unknown.

## Conclusion

SP has failed in Malawi and should no longer be used as a treatment for uncomplicated malaria. Its continued role in intermittent presumptive therapy in pregnancy programmes needs to be evaluated. In this study, AL was highly effective, even with unsupervised dosing and was well tolerated. Adherence to the six-dose regime was good and treatment was effective, even in those who did not properly comply with the dosing schedule. Self-reported adherence appears to be an unreliable measure of adherence in this population and may lead to over-estimation of the true level of adherence. This study provides strong reassurance of the effectiveness of AL as it is rolled out across sub-Saharan Africa.

## Conflict of interests

PAW and SAW were unpaid members of the MMV-GSK product development team for chlorproguanil-dapsone-artesunate. None of the other authors declare any conflicts of interest.

## Authors' contributions

The study was conceived and designed by DGL, PAW, SAW and MEM. The study was set up and run by DJB, DW, NK and PC. Laboratory analyses were performed by JM, DAH, DJB and DW. DJB, MM and DGL analysed the data. DJB, DGL, PAW and MEM wrote the paper. All authors read and approved the final manuscript.

## Funding

This work was funded by a grant from the Department for International Development, UK (Project number: RES 8074). The funders had no role in the study design or execution, or in the analysis and presentation of the data. GlaxoSmithKline provided the CPD medication without charge.
